# Cognitive traces of life kinetic exercise in puberty sedentary subjects

**DOI:** 10.1371/journal.pone.0322778

**Published:** 2025-05-09

**Authors:** Murat Sarikabak, Sezgin Hepsert, Ahmet Dönmez, Cengiz Baykara, Hasip Cana, Mert Ayranci, Hatice Aslı Çelebioğlu

**Affiliations:** 1 Faculty of Sport Sciences, Bartın University, Bartın, Türkiye; 2 Department of Physical Education and Sports, Fırat University, Institute of Health Sciences, Elazığ, Türkiye; 3 Faculty of Sport Sciences, Alanya Alaaddin Keykubat University, Antalya, Türkiye; 4 Ministry of National Education, Sakarya, Türkiye; 5 Sakarya University of Applied Sciences, Sapanca Vocational School of Tourism, Sakarya, Türkiye; 6 Faculty of Sport Sciences, Hitit University, Çorum, Türkiye; 7 Ministry of National Education, Manisa, Türkiye; Ordu University, TÜRKIYE

## Abstract

In this study, it was aimed to reveal the effect of regular life kinetic exercises on the concepts of motivation and imagery in sports in puberty period individuals. In the study in which “pre-test-post-test control group design” was used, 44 students (experiment = 22, control = 22) who were continuing their education in a state secondary school and selected by appropriate sampling method participated. The “Personal Information Form”, “Motivation Scale in Sport” and “Imagery Inventory in Sport” were used as data collection tools. The collected data were analysed using repeated measures analysis of variance. When the results were analysed, it was determined that there were statistically significant differences in the pre-test and post-test mean scores of motivation and imagery in sport. As a result, it can be stated that the life kinetic exercise protocol applied for 8 weeks in sedentary individuals positively predicted motivation and imagery parameters in sports. This result indicates that the related psychological phenomena, which are as important as sport performance in sports, can be positively affected by cognitive and motoric exercise combinations.

## Introduction

The brain serves as the foundation of human behavior, yet it undergoes structural and functional changes as a result of the behaviors it generates. This dynamic interplay between brain structure and function forms the neural basis of cognition, learning and adaptability [1]. It has been suggested that the human brain continues to develop and be shaped by experiences from childhood through adulthood [2]. Green and Bavelier argue that humans engage in some form of learning in nearly every context [3]. Substantial evidence from psychological research demonstrates that individuals improve in almost every task when exposed to appropriate stimulu, spanning perceptual learning [4] motor learning [5] and cognitive learning [6]. A key question arises: can Life Kinetic (LK) exercises serve as an effective stimulus for cognitive change, particularly in early adolescence? While numerous studies have explored this question from a motor development perspective across various sample groups [7–9], There is a notable scarcity of research investigating cognitive process (specifically imagery and motivation in sports) through multifaceted. This gap underscores the originality and significance of the present study.

Developed in Germany, LK is based on structuring new neural networks and enhancing the neurophysiological learning process [10]. This system cognitive training with motor coordination exercises, emphasizing the execution of multiple simultaneous movements [11]. To prevent the mere memorisation of movements patterns and to sustain cognitive engagement, stimuli. Notably, the threshold for effective engagement is set at approximately 60% accuracy in task performance. [12]. Host Lutz, the creator of LK, has consistently emphasized its necessity for both motor and cognitive health [13]. Within this framework the concepts of imagery [14] and motivation [15], both of which have been widely examined in cognitive research are considered key indicators of cognitive well-being.

Weinberg and Gould define imagery as “the process of mentally preparingoneself by visualising anticipated stimuli based on prior or newly acquired experiences “[16]. Expanding on this definition, Kızıldağ and Tiryaki describe imagery as “a process in which athletes visualize themselves performing at their best, thereby transforming mental rehearsal into actual performance” [17]. Meanwhile, motivation plays a fundamental role in learning, academic achievement, and sustained in or discontinue exercise and sports, particularly among sedentary populations [18]. Given its role in maintaining participation, motivation much like imagery is crucial for fostering long-term adherence to sports, regardless of an individual’s goals. Furthermore, these two psychological constructs share a common function in shaping and directing behavior [19].

A review of the literature highlights that motivation and imagery in sports are critical cognitive parameters for analyzing individual performance. These concepts have been extensively examined across diverse sample groups in previous studies, typically within experimental research design. In recent years, there has been growing interest in cognitive function during adolescence [20,21]. This is largely due to the recognition that puberty represents a critical period for the formation and refinement of an individuals’S cognitive map. Additionally, cognitive maturation and the emergence of abstract thinking skills during this stage [22] make adolescent populations particularly relevant for cognitive research. Existing studies on this population suggest that LK exercises have the potential to enhance cognitive function [23,24]. However, due to the limited number of available studies, scholars emphasize the need for further empiricial evidence [25,26]. In this context, the present study aims to examine the effects of regular LK exercises on motivation and imagery in sports among adolescents.

## Methods

### Research model

In this research, “pretest-posttest control group design”, one of the true experimental models, was employed. This design, widely used in social sciences research due to its methodological rigor and is frequently preferred in experimental research [27].

### Sample size

In this study, which examined changes in the measurement results obtained at two different time points (pre-test and post-test) from participants who did and did not perform Life Kinetic exercises, the sample size was determined using a power analysis. Since no directly comparable study was available in the literature as a reference, the minimum required sample size was calculated based on the effect sizes recommended by Cohen [28]. The sample size calculation was performed using the following parameters: effect size f = 0.40, type 1 error = 0.05, power of the test = 0.80, number of groups = 2, repetitions = 2, correlation between repeated measurements = 0.5 and de-sphericity correction = 1. As a result of the priori power analysis, the required sample size was determined to be 40 participants in total. However, to account for potential data loss, a 10% increase in the total sample size was applied, as recommended in the literature [29], resulting in a final sample of 44 participants. This adjustment increased the statistical power of the test to 0.84 in the post-hoc evaluation. The a priori power analysis for sample size determination was conducted using G*Power 3.1.9.7 (Franz Faul, UnversitatKiel, Germany).

### Research group

In the 2023–2024 academic year, a total of 44 students from a public secondary school in Elazığ province of Turkey, were selected through convenience sampling to participate in the study. These students were randomly assigned to either the experimental group (n = 22) or the control group (n = 22). Eligibility criteria for participation included not being regularly engaged in any sports activity and having the physical and cognitive capacity to perform the Life Kinetic exercises. Additionally, before the commencement of the experimental protocol, participants were informed about the exclusion criteria, which included engaging in physical exercise during the study period, voluntarily withdrawing from the study, or any other unforeseen reasons leading to discontinuation.

**Fig 1:**
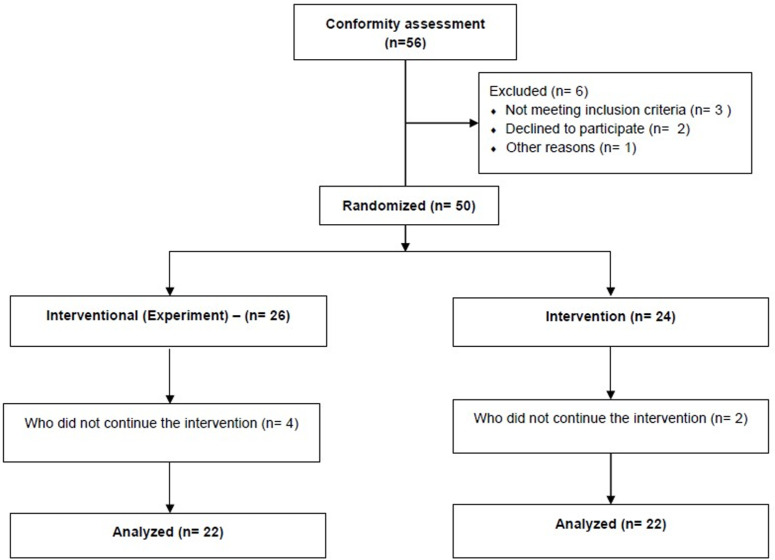
Flow chart of study design.

According to the descriptive statistics presented in Table 1, 50% (n = 22) of the participants were in the experimental group, while the remaining 50% (n = 22) were in the control group. Additionally, in both groups, 63.6% (n = 14) of the participants were female, and 36.4% (n = 8) were male. Regarding age distribution, the mean age of the experimental group was 12.95 ± 0.36, while the mean age of the control group was 12.59 ± 0.50. The overall mean age for the entire sample was 12.77 ± 0.48. The research flowchart is presented in Fig 1.

**Table 1:** Descriptive statistics about the participants.

Groups	Gender	n	%	${\overset{―}{\text{X}}}_{\text{age}}$	${\overset{―}{\text{X}}}_{\text{age}}$
Experimental	Girl	14	63.6	12.95 ± .36	12.77 ± .48
	Boy	8	36.4		
	Total	22	100.0		
Control	Girl	14	63.6	12.59 ± .50	
	Boy	8	36.4		
	Total	22	100.0		

### Control group intervention process

Participants randomly assigned to the control group did not engage in any mental or physical activity throughout the 8-week study period. During the pre-study informational session, participants were explicitly informed that any violation of this rule would result in exclusion from the study. Additionally, their written informed consent was obtained prior to participation.

### Life kinetic exercises applied to the experimental group

The life kinetic exercise protocol to be applied to the experimental group covers 8 weeks (3 days a week). To ensure standardisation in the study, the training sessions were performed on Mondays, Wednesdays, and Fridays between 15.00–17.00 hours in an indoor sports hall, between 18.03.2024 and 10.05.2024.

**Fig 2:**
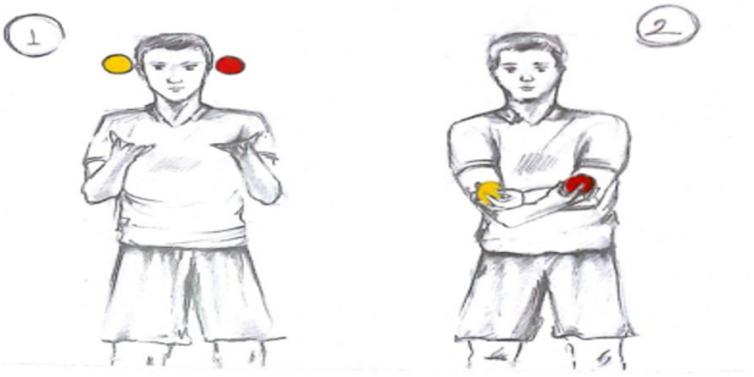
Throwing balls straight up in the air and catching them diagonally.

**Fig 3:**
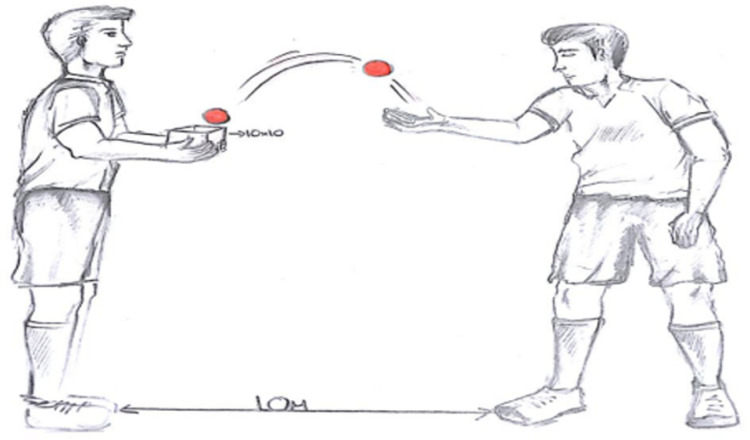
Throwing a ball at a target with eyes closed.

**Fig 4:**
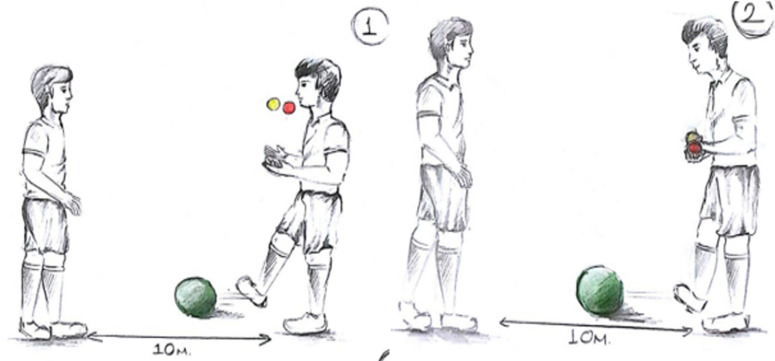
Counting exercise in the form of adding 5 to zero and subtracting 1 while making an in-foot pass and throwing and catching balls in the hand.

**Fig 5:**
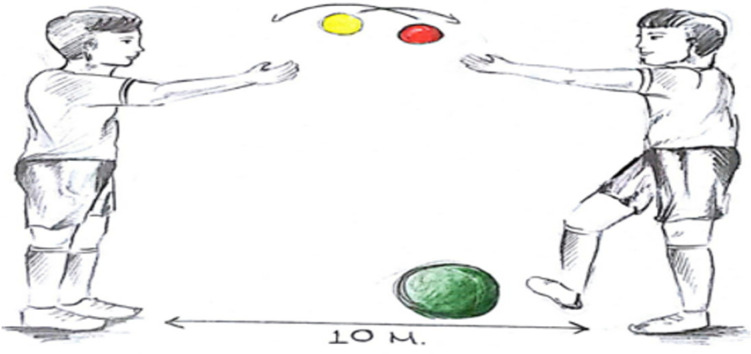
Exercise of changing the life kinetic balls in the hand while making an in-foot pass.

**Fig 6:**
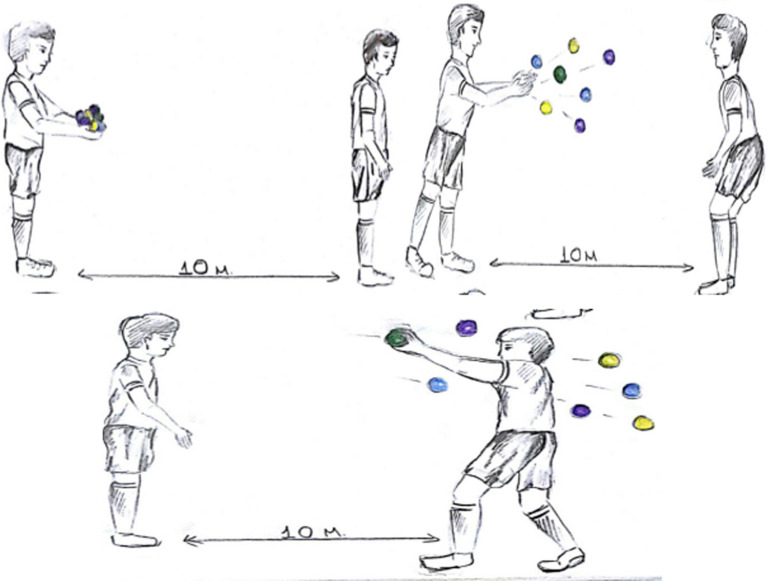
Exercise to catch the target ball.

**Fig 7:**
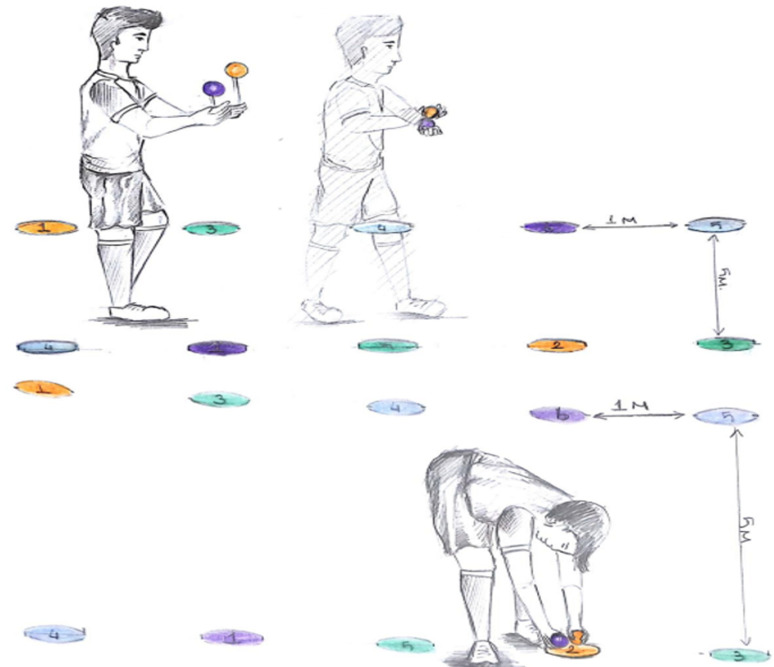
Exercise to find the right color and numbered circle.

The applied training protocol [30]:

1. ** Throwing balls in the hand straight up to the air and catching them diagonally:** Each subject was given 2 small life kinetic balls. The subjects were asked to throw the balls into the air at equal distances parallel to each other and to catch the balls by crossing their hands while the balls were falling. At this time, the ball in the right hand passed to the left hand and the ball in the left hand passed to the right hand. After the balls were held with crossed hands, this time the balls were thrown into the air while keeping the crossed position of the hands intact, and the balls that fell were held again by bringing the hands back to their original position and making them as straight as possible (Fig 2).

1. ** Throwing a ball at a target with eyes closed:** During this exercise, two subjects take opposite positions with a distance of 5 metres between them. One of the subjects holds a 10x10 cm box in his/her hand and the other subject tries to throw small life kinetic balls into the box. Before each throw, the subject opens his/her eyes, looks at the box and determines the required throw distance and route in his/her mind, then closes his/her eyes and performs the throw (Fig 3).

1. ** Counting exercise in the form of adding 5 to zero and subtracting 1 while making an in-foot pass and throwing and catching balls in the air:** During the exercise, the subjects take a mutual position with a distance of 5 metres between them. At the same time, while making an in-foot pass, they throw the life kinetic balls in their hands straight into the air and catch them diagonally, and a cognitive exercise is added to this. While performing these two actions at the same time, the subjects count by adding 5 and subtracting 1. For example, the subject first says 0, then 5, then 4, then 9, then 8, and continues the exercise (Fig 4).

1. **The exercise of changing the life kinetic balls in the hand during a mutual in-foot pass:** Two subjects take a position 10 metres apart. During the in-foot pass, the subjects exchange the balls in their hands by throwing them to each other at the same time. The subjects have to focus on both the ball coming from the air and the ball coming from the ground (Fig 5).

1. ** Exercise to Catch the Target Ball:** Two subjects are positioned 10 meters apart. One subject holds a total of 7 balls, consisting of 3 pairs of balls (each pair in a different color) and one single ball of a distinct color. The second subject stands 5 meters away with their back turned. Upon hearing the command to turn from the first subject, the second subject turns around. At that moment, the first subject throws all the balls into the air. The objective for the second subject is to catch the single colored ball (Fig 6).

1. ** Exercise to find the correct colour and numbered circle:** In this exercise, the subject walks along two parallel lines while navigating between six numbered circles (1–6) of four different colors, which are arranged on the floor in a complex pattern. As the subject moves, they must simultaneously throw Life Kinetic balls into the air and catch them diagonally, ensuring continuous engagement of motor coordination. The objective of the exercise is to identify the correct target circle based on a predetermined color-number pairing system and drop the balls onto the corresponding circle. For example, an orange pair (1) corresponds to the yellow circle (2), a green pair (2) corresponds to the yellow circle (4), a red pair (3) corresponds to the red circle (5), and a blue pair (4) corresponds to the blue circle (6). This exercise simultaneously challenges cognitive processing, motor coordination, and decision-making skills, requiring the subject to accurately match the correct pair while maintaining movement control and ball coordination. (Fig 7).

### Data collection tools

In addition to the personal information form, “Sport Motivation Scale II” and “Sports Imagery Questionnaire” were used as data collection tools in the study.

### Personal information form

The personal information form prepared by the researchers consisted of 2 questions to learn the personal information about gender and age of the individuals participating in the study.

### Sport motivation scale

The scale developed by Pelletier et al. was a revised version of the scale developed by the same authors in 1995 [31]. The measurement tool was revised as Motivation in Sport Scale-II with 18 items and adapted to Turkish culture by Öcal and Sakallı [32]. The measurement tool revealed intrinsic motivation, external motivation and amotivation, respectively, in sports environments. The Cronbach Alpha reliability coefficients of the scale, which was a 7 Likert scale, were calculated as.72 for intrinsic motivation,.72 for external motivation and.76 for amotivation [32]. The results related to the scale were presented in Table 2.

**Table 2:** The results related to the Sport Motivation scale.

		Factors	Skewness	Kurtosis	Cronbach α
Sport Motivation ScaleI-II	Pre-Test	Intrinsic Motivation	-.421	-.217	.705
		External Motivation	-.152	-.925	.822
		Amotivation	.893	.491	.650
	Post-Test	Intrinsic Motivation	-.460	.063	.701
		External Motivation	.049	-.671	.784
		Amotivation	.393	-.306	.610

The results in Table 2 showed that the normality values of the measurement tool vary within the range of ± 2 and these values were found to be suitable for normal distribution [33]; Cronbach Alpha coefficients were found to be above acceptable values [34].

### Sport imagery questionnaire

The Imagery in Sport Inventory developed by Hall, Mack, Paivio, and Hausenblas [35] and the Turkish reliability and validity study was conducted by Kızıldağ and Tiryaki [17]. The inventory, which originally consisted of 5 sub-dimensions and 30 items, was revised to 4 sub-dimensions and 21 items as a result of the study. The sub-dimensions of the 7-point Likert type scale were respectively: Cognitive Imagery, Motivational Specific Imagery, MotivationalGeneral-Arousal, Motivational General-Mastery. The Cronbach-Alpha reliability coefficients calculated for the sub-dimensions of the inventory were found to be.81 for the “Cognitive Imagery” sub-dimension,.80 for the “ Motivational Specific Imagery “ sub-dimension,.71 for the “Motivational General Arousal” sub-dimension and.59 for the “Motivational General Mastery” sub-dimension [17]. The results related to the scale were presented in Table 3.

**Table 3:** Results related to sports imagery scale.

		Sub-scales	Skewness	Kurtosis	Cronbach α
Sports İmagery Scale	Pre-Test	Cognitive Imagery	-.063	.190	.689
		Motivational Specific Imagery	.035	-.881	.745
		Motivational General-Arousal	-.430	.603	.630
		Motivational General Mastery	-.361	.011	.628
	Post-Test	Cognitive Imagery	-.259	-.988	.706
		Motivational Specific Imagery	.011	-.843	.786
		Motivational General-Arousal	.191	-.199	.636
		Motivational General Mastery	-.638	.472	.605

The results in Table 3 showed that the normality values of the measurement tool vary within the range of ± 2 and these values were found to be suitable for normal distribution [33]; Cronbach Alpha coefficients were found to be above acceptable values [34].

### Data collection

The research data were collected by the researcher in the spring term of 2023–2024 academic year. Firstly, pen and paper tests were applied to the participants to reveal the pre-tests, and then 8-week Life Kinetic exercises were applied. At the end of the application process, the pen and papertests applied at the beginning of the study were applied again and post-tests were taken and the study was completed.

### Ethical aspects of the research

To examine the scientific and ethical appropriateness of the research, written permission was obtained from the local ethics committee and the Ministry of National Education, Elazığ Provincial Directorate of National Education. The research was conducted according to the Declaration of Helsinki, and the participants and their families were informed about the aim, structure, and procedure of the research. Written informed consent forms were also obtained from the participants and their parents.

### Data analysis

The data obtained from the research was transferred to SPSS 25.0 (IBM, Armonk, NY, USA) package programme with numerical coding. Firstly, descriptive statistics, then the reliability coefficients of the measurement tools were calculated and the normal distribution assumptions for the data for the analyses to be applied were examined through skewness and kurtosis values. As a result of the procedures, it was determined that the values were within the range of ± 2. Literature [33] supported that these values have a normal distribution. Repeated Measures ANOVA was used in statistical analyses and the significance level was accepted as p < .05. Additionally, GraphPad Prism 8 was used to prepare the figures.

## Results

**Fig 8:**
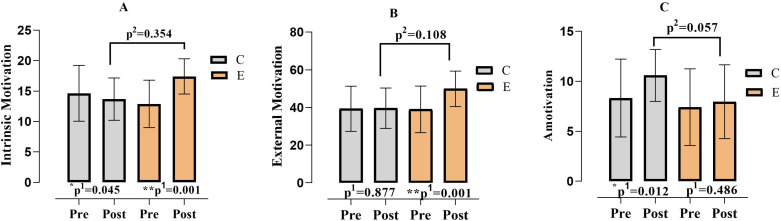
Comparison of participants’ sports motivation values according to groups and measurement times. p^1^; within group, p^2^; posttest comparison between groups, C; Control, E; Experiment, *;p < 0.05, **;p < 0.01.

When Fig 8. was examined, it was determined that the values of participants’ intrinsic motivation (F = .880; p = .354; η^2^ = .021), external motivation (F = 2.705; p = .108; η^2^ = .247) and amotivation (F = 3.828; p = .057; η^2^ = .084) did not differ according to the experimental and control groups. It was determined that the mean values of participants’ intrinsic motivation (F = 20.856; p = .000; η^2^ = .332), external motivation (F = 15.445; p = .000; η^2^ = .269) and amotivation (F = 6.158; p = .017 η^2^ = .128) pre-measurement and post-measurement differed according to time. Finally, while a significant difference was obtained in the group*time interaction in intrinsic motivation (F = 49.363; p = .000; η^2^ = .540) and external motivation (F = 13.744; p = .000; η^2^ = .247), no significant difference was obtained in the group*time interaction in amotivation (F = 2.313; p = .136; η^2^ = .052).

**Fig 9:**
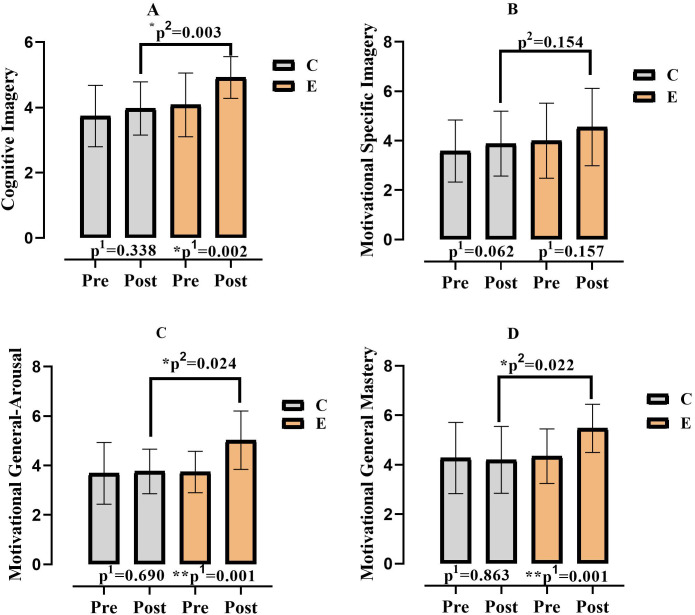
Comparison of participants’ imagery values according to groups and measurement times. p^1^; within group, p^2^; posttest comparison between groups, C; Control, E; Experiment, *;p < 0.05, **;p < 0.01.

When Fig 9 was examined, it was found that the participants’ cognitive imagery (F = 9.643; p = .003; η^2^ = .187), motivational general arousal (F = 5.501; p = .024; η^2^ = .116) and motivational general mastery (F = 5.656; p = .022; η^2^ = .119) values differed according to the experimental and control groups; while the motivational specific imagery values (F = 2.105; p = .154; η^2^ = .048) did not differ according to the experimental and control groups. It was found that the participants’ cognitive imagery (F = 12.584; p = .001 η^2^ = ,231), motivational specific imagery (F = 4.381; p = .042; η ^2^ = .094), motivational general arousal (F = 20.293; p = .000 η ^2^ = .326) and motivational general mastery (F = 4.749; p = .035; η^2^ = .102) pre-measurement and post-measurement means differed according to time. Finally, while a significant difference was found in the group*time interaction of motivational general arousal (F = 15.834; p = .000 η^2^ = .274) and motivational general mastery (F = 6.225; p = .017; η^2^ = .129), no significant difference was found in the group*time interaction of cognitive imagery (F = 4.032; p = .051; η^2^ = .088) and motivational specific imagery (F = .389; p = .536 η^2^ = .009).

## Discussion and conclusion

Life Kinetic (LK) exercises, which have the potential to stimulate both cognitive and motor skill development, represent an appealing training protocol for both sedentary and trained individuals. Positioned within the framework of multifaceted development, LK has gained recognition in the literature over the past two decades, advocating for biochemical brain adaptation through external stimuli to facilitate new learning. Aligned with this perspective, the present study aimed to examine the effects of a regular LK exercise intervention on motivation and imagery in sports.

When the research results were examined, it was determined that the intervention protocol applied did not produce a significant intergroup effect on the motivation variable in sports; however a significant within-group difference in favor of the experimental group was observed over time. Motivation in sports participation is a crucial concept for the detailed analysis of youth sports. On the other hand, this concept is generally examined within the framework of causality, which drives individuals to physical activities [36,37] and there are limitations in assessing the predictive power of experimental protocols such as LK. “The brain is the source of behaviour and can be changed by behaviour itself” [38], this compelling statement effectively summarizes the functional mechanism of brain plasticity. Although brain plasticity is considered as a critical factor during adolescence, it is possible that the excitability and adaptability of brain plasticity, which can be stimulated through cognitively demanding activities, may increase with repeated exposure to structured behavioral protocols [39]. Brain plasticity and motivation are interdependent mechanisms that reinforce each other. Motivation strengthens synaptic connections in the brain, enhancing plasticity, while greater plasticity, in turn, positively influences motivation [40].

In this context, LK exercises play a crucial role in brain architecture, contributing to brain plasticity and an increase in synaptic connections, provided they are applied consistently and systematically [41,42]. Motivation generally functions as a driving force behind behavior activation [43] and can be shaped through repeated exposure to novel stimuli in behavioral processes [44]. The literature supports the notion that greater sports experience and longer training duration are predictors of motivation in individuals during early adolescence [45,46]. Furthermore, environmental stimuli, meditation, and cognitive processes can induce changes in the brain through activity-dependent plasticity, which, in turn, impacts healthy living and learning [47,48]. Sports activities can facilitate measurable neurobiological changes, creating an optimal foundation for brain development [49]. Given this perspective, it can be inferred that the LK exercise protocol has the potential to serve this function effectively.

The findings of this study, interpreted in light of the existing literature, suggest that regular engagement in LK exercises has the capacity to positively influence participants’ cognitive-motivational behaviors. However, various underlying mechanisms may contribute to this increase in motivation. According to Bandura’s (1997) self-efficacy theory, successfully completing a given task fosters positive changes in self-confidence. Since LK exercises continually introduce new and varied motor-cognitive challenges, they may enhance motivation by reinforcing a sense of achievement [50].

Similarly, based on Csikszentmihalyi’s (2014) flow theory, motivation is expected to increase when individuals are fully immersed in an activity and experience an optimal level of challenge. Given that LK exercises require sustained attention and cognitive engagement, they may facilitate the flow state, thereby promoting motivational gains [51].

While intergroup significance was observed in the cognitive imagery, motivational general arousal, and motivational general mastery parameters, no significant difference was found in the motivational specific imagery variable. Furthermore, the intervention protocol significantly predicted imagery in favor of the experimental group. Although imagery has been extensively examined in performance-oriented studies involving athletes [52,53], it is also a crucial phenomenon in the daily lives of sedentary individuals, particularly in terms of self-evaluation and encouragement toward sports participation. There exists a bidirectional interaction between movement and perceptual experiences—perceptions influence movements, while movements, in turn, shape perceptual experiences. This interplay plays a fundamental role in the development of both motor and cognitive processes and is believed to be stored in long-term memory [54]. Long-term memory is closely linked to motor action and visual-motor control [55]. Within this framework, it has been suggested that temporal information (stimuli) can influence long-term memory, leading to changes in imagery, a key cognitive variable [56]. Moreover, most motor skills encompass both physical and cognitive components, and not only physical practice but also cognitive interventions can enhance imagery [57].

Frank (2014) demonstrated that three days of imagery practice, or a combination of imagery training and physical preparation, resulted in greater swing performance improvements in novice golfers compared to a control group with no practice. Since LK integrates both physical and cognitive elements, its predictive effect on imagery can be attributed to this characteristic. This perspective is further supported by Frank et al. (2014), who found that motor imagery training led to positive developments in the mental representation structure of an athlete sample [58]. Additionally, previous research suggests that perceptual-cognitive abilities are significantly higher in individuals engaged in sports compared to sedentary individuals [59,60]. In this regard, the finding that mean imagery scores were lower in the control group (comprising sedentary individuals) than in the experimental group suggests that the 8-week LK exercise intervention effectively enhanced imagery scores in sedentary individuals.

As a result, it can be stated that the 8-week LK exercise protocol applied to sedentary individuals positively predicted motivation and imagery parameters in sports. This finding indicates that psychological factors, which are just as important as performance in sports, can be enhanced through a combination of cognitive and motor exercises. Experimental research plays a crucial role in generating new insights on this topic. In particular, incorporating time-interval assessments into future study designs would help to provide more specific findings regarding the long-term effects of such interventions on both individuals and target sample groups.

## Limitations

This study offers valuable contributions to understanding the effects of Life Kinetic (LK) exercises on cognitive and motivational parameters; however, it has certain limitations that warrant consideration. The sample size of 44 participants, while adequate for statistical analysis, may limit the broader applicability of the findings to other populations or age groups outside of early adolescence. Additionally, the use of convenience sampling may influence the representativeness of the sample, potentially introducing selection bias. The study’s reliance on self-reported measures, though validated, carries the risk of response biases, such as social desirability. Furthermore, the lack of follow-up assessments restricts insights into the long-term sustainability of the observed effects. Addressing these limitations in future research could enhance the robustness and applicability of the results.

## Generalizability of findings

The findings of this study provide significant insights into the potential of LK exercises to enhance cognitive and motivational outcomes; however, their generalizability should be interpreted with caution. The study’s focus on a homogenous group of sedentary adolescents from a specific geographical and cultural context limits the direct application of these results to other populations, such as different age groups or active individuals. Additionally, the controlled experimental conditions may not fully reflect the complexities of real-world environments. Future research could benefit from broader and more diverse sampling strategies, as well as the inclusion of varied contexts, to further validate and extend the generalizability of these findings.

## Supporting information

S1 Data Data File. (XLSX)

S2 Checklist CONSORT-2010-Checklist. (DOC)
